# Predictors of Intrahospital Mortality in Aneurysmal Subarachnoid Hemorrhage after Endovascular Embolization

**DOI:** 10.3390/medicina60071134

**Published:** 2024-07-15

**Authors:** Valentina Opancina, Nebojsa Zdravkovic, Slobodan Jankovic, Dragan Masulovic, Elisa Ciceri, Bojan Jaksic, Jasmin J. Nukovic, Jusuf A. Nukovic, Miljan Adamovic, Miljan Opancina, Nikola Prodanovic, Merisa Nukovic, Tijana Prodanovic, Fabio Doniselli

**Affiliations:** 1 Department of Radiology, Faculty of Medical Sciences, University of Kragujevac, 34000 Kragujevac, Serbia; 2 University Clinical Center Kragujevac, 34000 Kragujevac, Serbia; 3 Diagnostic Imaging and Interventional Neuroradiology Unit, Department of Neurosurgery, Fondazione IRCCS Istituto Neurologico Carlo Besta, 20133 Milan, Italy; 4 Department of Medical Statistics and Informatics, Faculty of Medical Sciences, University of Kragujevac, 34000 Kragujevac, Serbia; 5 Department of Pharmacology and Toxicology, Faculty of Medical Sciences, University of Kragujevac, 34000 Kragujevac, Serbia; 6 Department of Radiology, Medical Faculty, University of Belgrade, 11120 Belgrade, Serbia; 7 Faculty of Medicine, University of Kosovska Mitrovica, 11000 Belgrade, Serbia; 8 Department of Radiology, General Hospital Novi Pazar, 36300 Novi Pazar, Serbia; 9 Faculty of Pharmacy and Health Travnik, University of Travnik, 72270 Travnik, Bosnia and Herzegovina; 10 Faculty of Medical Sciences, University of Kragujevac, 34000 Kragujevac, Serbia; 11 Pharmacy Institution “Zdravlje Lek”, Prvomajska 100, 11000 Belgrade, Serbia; 12 Faculty of Medicine, Military Medical Academy, University of Defense, 11000 Belgrade, Serbia; 13 Department of Surgery, Faculty of Medical Sciences, University of Kragujevac, 34000 Kragujevac, Serbia; 14 Department of Pediatrics, Faculty of Medical Sciences, University of Kragujevac, 34000 Kragujevac, Serbia; 15 Department of Neuroradiology, Fondazione IRCCS Istituto Neurologico Carlo Besta, 20133 Milan, Italy

**Keywords:** intracranial aneurysm, subarachnoid hemorrhage, endovascular embolization, risk factors, mortality

## Abstract

*Background and Objectives:* Aneurysmal subarachnoid hemorrhage (ASAH) is defined as bleeding in the subarachnoid space caused by the rupture of a cerebral aneurysm. About 11% of people who develop ASAH die before receiving medical treatment, and 40% of patients die within four weeks of being admitted to hospital. There are limited data on single-center experiences analyzing intrahospital mortality in ASAH patients treated with an endovascular approach. Given that, we wanted to share our experience and explore the risk factors that influence intrahospital mortality in patients with ruptured intracranial aneurysms treated with endovascular coil embolization. *Materials and Methods:* Our study was designed as a clinical, observational, retrospective cross-sectional study. It was performed at the Department for Radiology, University Clinical Center Kragujevac in Kragujevac, Serbia. The study inclusion criteria were ≥18 years, admitted within 24 h of symptoms onset, acute SAH diagnosed on CT, aneurysm on DSA, and treated by endovascular coil embolization from January 2014 to December 2018 at our institution. *Results:* A total of 66 patients were included in the study—48 (72.7%) women and 18 (27.3%) men, and 19.7% of the patients died during hospitalization. After adjustment, the following factors were associated with in-hospital mortality: a delayed ischemic neurological deficit, the presence of blood in the fourth cerebral ventricle, and an elevated urea value after endovascular intervention, increasing the chances of mortality by 16.3, 12, and 12.6 times. *Conclusions:* Delayed cerebral ischemia and intraventricular hemorrhage on initial head CT scan are strong predictors of intrahospital mortality in ASAH patients. Also, it is important to monitor kidney function and urea levels in ASAH patients, considering that elevated urea values after endovascular aneurysm embolization have been shown to be a significant risk factor for intrahospital mortality.

## 1. Introduction

Aneurysmal subarachnoid hemorrhage (ASAH) is defined as “bleeding in the subarachnoid space caused by the rupture of a cerebral aneurysm” [1,2,3]. Epidemiological ASAH data suggest that 9 out of 100,000 people in the US and almost 600,000 people worldwide experience it annually [4]. About 11% of people who develop ASAH die before receiving medical treatment, and 40% of patients end up dying within four weeks of being admitted to the hospital [5]. ASAH has a great impact on morbidity, so up to 30% of surviving patients show some degree of disability [5].

After providing emergency medical assistance, selecting an adequate diagnostic modality, and establishing a diagnosis, the next step is the treatment of brain injury caused by the aneurysm bleeding [6]. This is achieved by excluding the source of the bleeding from the circulation, whereby the speed of response in terms of the treatment reduces the mortality of these patients [7]. More precisely, if the exclusion of the cerebral aneurysm from the circulation is performed on the second day after the subarachnoid hemorrhage, the outcome of such patients is better than if it is done later [8]. An aneurysm that caused an ASAH can be treated with an open surgical method by placing a metal clip whose role is to occlude the neck of the aneurysm [9]. However, the treatment option is decided with interdisciplinary consent. Endovascular embolization (EE) is a minimally invasive method and involves “performing the procedure using a catheter under fluoroscopic guidance from the entry point in the artery (usually the femoral artery in the groin) to the parent artery where the aneurysm is” [10]. Two randomized trials that compared endovascular treatment with a surgical approach in cerebral aneurysms after their rupture, the International Subarachnoid Aneurysm Trial (ISAT) and the Barrow Ruptured Aneurysm Trial, came to the conclusion that the patients treated with the endovascular method had better functional outcomes in the first year after treatment. It should be noted that the authors also stated that the complete occlusion rate after endovascular treatment was only about 50% in the long term and frequent retreatment may be required [10,11,12,13,14]. Significant morbidity or mortality take place in 8% to 23% of patients with rebleeding [15]. The most important period is the first six hours after the initial bleeding, as up to 90% of rebleeding occurs in that time frame [16,17]. Also, the risk groups of patients for this complication include those who have a more severe form of ASAH and a large aneurysm, as well as those who wait for aneurysm embolization for a long time [18].

Still, data on intrahospital mortality for treated ruptured intracranial aneurysms vary from 8.3% to 66.7% [19]. A recent study explored mortality risk factors in ASAH patients with subarachnoid hemorrhage hospitalized in an Intensive Care Unit (ICU) and revealed mortality of 40% and the following predictors: aspiration pneumonia, septic shock, a CT-diagnosed midline shift, inter-hospital transfer, and hypernatremia (all during the first 72 h in the ICU). It was also stated that endovascular or surgical treatment performed within the first 72 h was a good prognostic factor [20]. There are limited data on single-center experiences analyzing intrahospital mortality in ASAH patients treated with an endovascular approach. Given that, we wanted to share our experience and explore the risk factors that influence intrahospital mortality in patients with ruptured intracranial aneurysms treated with endovascular coil embolization.

## 2. Materials and Methods

Our study was designed as a clinical, observational, retrospective cross-sectional study. It was performed at the Department for Radiology, University Clinical Center Kragujevac in Kragujevac, Serbia. The study was approved by the Institutional Ethics Board and conducted according to the principles of the Helsinki Declaration.

The study inclusion criteria were ≥18-year-old patients, admitted within 24 h of symptoms onset, a first-time intracranial aneurysm rupture diagnosed by CT and digital subtraction angiography, and treated with endovascular coil embolization (EE) from January 2014 to December 2018 at our institution. We excluded patients who had been treated with EE previously due to an aneurysm rupture, who had CT scans with artefacts, incomplete medical documentation, hepatorenal dysfunction, or malignant disease, and pregnant women. The interventional procedure was performed by two neuroradiologists with expertise in the field, and the CT scans were assessed by three experienced neuroradiologists at our institution. Most of the procedures were done within 72 h from the ictus. The indication and timing of the treatment were decided by a decision of a multidisciplinary board, including neurosurgeons, neurologists, neuroradiologists, and an interventional radiologist.

An ASAH was diagnosed on CT upon admission with the presence of blood in the subarachnoid space, while an aneurysm was diagnosed on DSA. Laboratory results that were taken in account were divided into three groups, depending on the day of the endovascular procedure: 24 to 48 h before the procedure, on the day when the endovascular procedure was performed, and 24 to 48 h after the procedure.

Our study explored the following variables from the patient files: socio-demographic data; the clinical picture prior to admission; scales: the Hunt and Hesse scale (HHS) and the Glasgow Coma Scale (GCS), both performed on admission [21], and the Fisher scale (FS) performed using the initial head CT scan [22]; the findings of the initial CT scan; a lumbar puncture and/or ventriculoperitoneal shunt; the application of mechanical ventilation after EE; the aneurysm features seen on DSA; the characteristics of the interventional procedure; hospitalization length, the time frame from admission to the hospital to the interventional procedure, and the duration of symptoms prior to admission; and laboratory analysis, which included the blood count, coagulation tests, and biochemistry analysis.

### Statistics

We used SPSS version 23 software (SPSS Inc., Chicago, IL, USA) to analyze the study data [22]. Descriptive statistics were used initially. In order to describe continuous variables, the mean and standard deviation (if there was normal distribution) or the median and interquartile range (if there was not normal distribution) were used. We displayed categorical variables using rates and percentages. In order to test the normality of the data distribution, the Kolmogorov–Smirnov test was performed. The Mann–Whitney test was used for continuous variables and contingency tables were used for categorical variables when the significance of differences in the study groups was explored. The influence of study variables on death outcome was checked by univariate logistic regression and later on with multivariate logistic regression with the use of a backward conditional method. The Hosmer–Lemeshow, Cox–Snell, and Nagelkerke tests were used to explore the quality of the multivariate logistic regression model.

## 3. Results

After applying the inclusion and exclusion criteria of the study, the study included and analyzed a total of 66 ASAH patients. Of these, 48 (72.7%) were women and 18 (27.3%) were men. The youngest patient was 30 years old and the oldest 80 years old, while the mean age of the patients was 54 years. All patients were treated with endovascular aneurysm embolization, of whom 33 developed cerebral vasospasm, 14 patients developed hydrocephalus, and 42 developed brain edema. Thirteen patients (19.7%) died during hospitalization.

Table 1 presents the main characteristics of the study population. The variables explored in the laboratory analysis of the study population are presented in Table 2.

The results of the univariate logistic regression for the death outcome are depicted in Table 3.

After adjustment, the following factors were associated with in-hospital mortality: a delayed ischemic neurological deficit, the presence of blood in the fourth cerebral ventricle, the length of hospitalization, and an elevated urea value after endovascular intervention (Table 4). The strength of the relationship prevailed similar to that after the univariate analysis. Also, there was no change in the direction of influence. The estimates of the coefficient of determination according to the Cox–Snell and Nagelkerke tests were 0.368 and 0.595, respectively. Also, the Hosmer–Lemeshow test demonstrated that the observed rate of intrahospital mortality matched the expected rate of the same phenomenon (χ^2^ = 6.770, *p* = 0.453). Within the multivariate logistic regression, the examination of the interactions between the variables did not yield significant results.

## 4. Discussion

Examining the death outcome in patients with ASAH who were treated with endovascular embolization, it was shown that four factors are related to this outcome; namely, a delayed ischemic neurologic deficit, hemorrhage in the fourth cerebral ventricle, the length of hospitalization, and elevated urea after endovascular embolization. It was shown that if a patient develops a delayed ischemic neurologic deficit, the chances of a fatal outcome increase by 16.3 times. The presence of blood in the fourth cerebral ventricle, which was visualized on the initial CT scan, increased the chance of a fatal outcome by 12 times. The statistical results showed that an increase in the length of hospitalization reduced intrahospital mortality by 0.9 times, or, better said, the surviving patients had a longer treatment time compared to the non-survivors, who died early on. An increase in the value of urea above the reference values after an endovascular procedure increased the chance of the patient’s death by 12.6 times.

So far, the following risk factors for death in patients with subarachnoid hemorrhage have been described in various studies: age, large aneurysms, cerebral ischemia as a consequence of vasospasm, vasospasm, a poor clinical picture on admission, brain edema, and intraventricular hemorrhage [23,24,25]. The intrahospital mortality of the patients with ASAH in one of the studies was 18% [26], which is close to our result. Another study with a smaller number of patients showed higher in-hospital mortality of 23% [27]. There were also studies with a significantly lower mortality rate (6%), but with a higher rate of poor clinical outcome (30%) that included severe disability, the vegetative state of the patient, and death [28].

It has also been shown that the presence of cerebral ischemia (*p* = 0.039), symptomatic vasospasm (*p* = 0.039), and pneumonia (*p* = 0.006) are connected with a poor outcome in ASAH patients after endovascular treatment [29].

Massive subarachnoid hemorrhage and massive intraventricular hemorrhage are strong prognostic factors for the occurrence of a lethal outcome in ASAH [27]. This can be explained by the fact that intraventricular hemorrhage is associated with the occurrence of numerous complications, such as ventriculitis, fever, and hydrocephalus, which also significantly increase the mortality rate [30]. Previous research has shown that the presence of blood in the fourth cerebral ventricle is prognostic of a poor outcome in patients with SAH [31], which supports our result, which speaks of a strong relationship with this diagnostic sign, as it increased the chances of death by 12 times. This result is very important because the initial brain CT is indispensable in all patients with suspected subarachnoid hemorrhage, and, therefore, special attention should be paid to the presence of blood in the fourth cerebral ventricle in the context of a poor prognostic factor.

A study of poor outcomes and primarily death in patients with aneurysmal subarachnoid hemorrhage showed that with the increasing age of the patients with ASAH, the risk of death and other poor outcomes increased [32]. Our results showed a significant relationship between patient age and mortality only in the univariate logistic regression, but not in the final model.

The role of cerebral vasospasm, both angiographic and symptomatic, a delayed ischemic neurological deficit, and delayed cerebral ischemia in the occurrence of death has been investigated, but conflicting results have been published. A delayed ischemic neurologic deficit was shown to be the cause of death in 36% of patients, while single associations between poor outcome and angiographic vasospasm, neurologic deterioration, and brain ischemia were demonstrated (*p* < 0.0001) [28]. Examining three entities within delayed cerebral ischemia proved that symptomatic vasospasm without the development of ischemia, symptomatic CVS and ischemia, as well as delayed ischemia without cerebral vasospasm, individually showed a significant relationship with the development of a fatal outcome (*p* = 0.095, *p* = 0.004, *p* = 0.000), while in the multivariate regression model they lost their significance [26]. The results of this study showed that a delayed ischemic neurological deficit was a strong predictor for in-hospital mortality. In this regard, it is necessary to pay extra attention to potential neurological deterioration in order to diagnose delayed ischemic neurological deficit in time and to prevent further progression and development of delayed cerebral ischemia, which is a known risk factor for death in subarachnoid hemorrhage, with drug therapy [23]. The reason for these conflicting results may be the constant progress of endovascular procedures used for the treatment of ruptured aneurysms, which have great benefits for patients [23,26].

Very few studies have so far examined laboratory results as a risk factor for the occurrence of death in ASAH. In our research, the results of the laboratory diagnostics showed the importance of elevated urea levels after endovascular embolization of a ruptured aneurysm as a strong predictor of death in these patients. It is important to monitor kidney function and creatinine and urea levels in ASAH patients, considering that patients at risk for developing renal failure have twice the chance of death [33]. Also, untreated renal dysfunction is the strongest predictor for in-hospital mortality in ASAH (*p* < 0.001), while acute kidney injury is a confirmed risk factor (*p* = 0.028) [33]. There are no clear pathophysiological mechanisms to explain renal damage in ASAH, but the influence of hemodynamic instability that develops after aneurysm rupture, as well as potential nosocomial infections or the onset of shock, is suspected [34]. Still, a recent study investigated the urea/creatinine ratio in non-traumatic subarachnoid hemorrhage patients and revealed that the ratio is a risk factor for increased intrahospital mortality in these patients. The authors proposed several mechanisms to explain this, including (i) a body stress response leading to unstable brain and kidney circulation and (ii) dehydration that happens early in subarachnoid hemorrhage due to consciousness disorder or dysphagia [35]. Other research investigated the urea/creatinine ratio in ASAH patients with a sample size of 66 patients, like our study. It was mentioned that the urea/creatinine ratio may be a potential marker for catabolism due to critical illness. Also, it was demonstrated that the ratio had increased early values as a risk factor for poor outcome after one year, while greater critical values were connected with DCI, as well as DCI-related infarctions [35]. Both of these studies investigated the urea/creatinine ratio and suggested further research on this marker, which is in concordance with our results. The role of angiography and contrast media use should also be investigated in further studies as a possible factor that can contribute to renal dysfunction. Further research on kidney function and creatinine and urea levels in ASAH patients is needed in order to explain pathophysiological mechanisms. We propose further studies analyzing intrahospital mortality in ASAH patients treated with an endovascular approach, including a larger sample size.

This study has some limitations. It was a unicentric study with a relatively small sample size. Due to the small sample, it was possible to simultaneously test only up to 10 variables in the multivariate logistic regression, which increased the chances of a statistical type 2 error.

## 5. Conclusions

From the obtained results, we can conclude that a delayed ischemic neurologic deficit is the strongest predictor of intrahospital mortality in ASAH patients. Also, it is important to pay attention to the intraventricular hemorrhage on the initial head CT scan, along with monitoring kidney function and elevated urea values after endovascular aneurysm embolization, since they have been shown to be a significant risk factor for intrahospital mortality in ASAH patients.

## Figures and Tables

**Table 1:** Characteristics of study population.

Risk Factors	Patients Alive (n = 53)	Death Outcome (n = 13)	*p* Value
Age (Mean ± SD, Median [IQR])	52.43 ± 9.964, 54 [17]	60.38 ± 12.725, 56 [21]	0.054
Age category (20–40/40–60/>60 years)	8/31/14 (15.1%/58.5%/26.4%)	1/6/6 (7.7%/46.2%/46.2%)	0.360
Gender (male/female, %/%)	13/40 (24.5%/75.5%)	5/8 (38.5%/61.5%)	0.312
Caffeine usage (no/yes)	23/30 (43.4%/56.6%)	5/8 (38.5%/61.5%)	0.747
Smoking (no/yes)	29/24 (54.7%/45.3%)	7/6 (53.8%/46.2%)	0.955
DM as comorbidity (no/yes)	51/2 (96.2%/3.8%)	12/1(92.3%/7.7%)	0.543
Hypertension as comorbidity (no/yes)	27/26 (50.9%/49.1%)	6/7 (46.2%/53.8%)	0.757
Clinical appearance on admission:			
Headache (no/yes)	8/45 (15.1%/84.9%)	0/13 (0%/100%)	0.135
Nausea (no/yes)	28/25 (52.8%/47.2%)	7/6 (53.8%/46.2%)	0.948
Vomiting (no/yes)	29/24 (54.7%/45.3%)	8/5 (61.5%/38.5%)	0.657
Altered conscience (no/yes)	21/32 (39.6%/60.4%)	5/8 (38.5%/61.5%)	0.939
Coma (no/yes)	49/4 (92.5%/7.5%)	11/2 (84.6%/15.4%)	0.378
Arterial blood pressure (hypotension/hypertension)	11/42 (20.8%/79.2%)	3/10 (23.1%/76.9%)	0.854
Neck rigidity (no/yes)	20/33 (37.7%/62.3%)	3/10 (23.1%/76.9%)	0.320
GCS (Mean ± SD, Median [IQR])	11.38 ± 3.065, 13 [5]	10.08 ± 4.291, 12 [7]	0.457
GCS (≤8/>8)	12/41 (22.6%/77.4%)	3/10 (23.1%/76.9%)	0.973
Duration of symptoms in hours, before admission (Mean ± SD, Median [IQR])	23.151 ± 25.582, 6 [22.75]	23.923 ± 38.300, 3 [37.5]	0.231
Duration of symptoms in hours, before admission (<6 h/6–12 h/12–24 h)	34/1/18 (64.2%/1.9%/34%)	7/3/3 (53.8%/23.1%/23.1%)	0.016
Time from onset of symptoms to EE, in hours (<2/2–4/4–6/6–12/>12 h)	10/19/9/2/13 (18.9%/35.8%/17%/3.8%/24.5%)	3/1/3/3/3 (23.1%/7.7%/23.1%/23.1%/23.1%)	0.085
Time from onset of symptoms to medication, in hours (Mean ± SD, Median [IQR])	38.481 ± 102.952, 6 [13.5]	24.154 ± 38.179, 4 [37.5]	0.342
Duration of fluoroscopy in EE (Mean ± SD, Median [IQR])	27.26 ± 6.346, 27 [10]	31.69 ± 6.33, 30 [11]	0.027 *
Aneurysm size (<5 mm/5–10 mm/11–25 mm)	16/30/7/0 (30.2%/56.6%/13.2%/0%)	2/6/5/0 (15.4%/46.2%/38.5%/0%)	0.095
Aneurysm location (ACI/ACM/ACA/ACP/AB)	22/7/19/2/3 (41.5%/13.2%/35.8%/3.8%/5.7%)	3/3/4/2/1 (23.1%/23.1%/30.8%/15.4%/7.7%)	0.396
Aneurysm height (Mean ± SD, Median [IQR])	7.108 ± 4.031, 6.72 [4.67]	8.634 ± 4.485, 7.08 [6.57]	0.211
Aneurysm width (Mean ± SD, Median [IQR])	5.816 ± 2.996, 4.45 [4.3]	7.079 ± 4.103, 6.29 [5.09]	0.314
Aneurysm neck (Mean ± SD, Median [IQR])	2.871 ± 1.004, 2.70 [1.23]	4.761 ± 4.424, 3.80 [4.56]	0.589
Cerebral vasospasm (no/yes)	29/24 (54.7%/45.3%)	4/9 (30.8%/69.2%)	0.122
Hydrocephalus (no/yes)	42/11 (79.2%/20.8%)	10/3 (76.9%/23.1%)	0.854
Brain edema (no/yes)	20/33 (37.7%/62.3%)	4/9 (30.8%/69.2%)	0.640
Intracerebral or intracerebellar hematoma (no/yes)	32/21 (60.4%/39.6%)	10/3 (76.9%/23.1%)	0.266
Intraventricular hemorrhage (no/yes)	30/23 (56.6%/43.4%)	0/13 (0%/100%)	0.000 *
Hemorrhage in IV ventricle (no/yes)	40/13 (75.5%/24.5%)	6/7 (46.2%/53.8%)	0.039
HHS (Mean ± SD, Median [IQR])	2.96 ± 1.255, 3 [2]	2.69 ± 1.182, 3 [2]	0.463
FS (Mean ± SD, Median [IQR])	3.09 ± 0.883, 3 [2]	3.85 ± 0.376, 3 [2]	0.004 *
Mechanical ventilation (no/yes)	39/14 (73.6%/26.4%)	2/11 (15.4%/84.6%)	0.000 *
Liquor evacuation with lumbar puncture (no/yes)	44/9 (83%/17%)	8/5 (61.5%/38.5%)	0.090
Ventriculoperitoneal shunt (no/yes)	47/6 (88.7%/11.3%)	10/3 (76.9%/23.1%)	0.268
Duration of hospitalization (Mean ± SD, Median [IQR])	32.207 ± 19.018, 30 [25]	19.385 ± 14.847, 17 [13]	0.005 *

*—statistically significant; Abbreviations: ACI—a.carotis interna, ACM—a.cerebri media, ACA—cerebri anterior, ACP—a.cerebri posterior, AB = a.basillaris, GCS—Glasgow Coma Scale, HHS—Hunt and Hesse scale, FS—Fisher scale, DM—Diabetes mellitus. The Mann–Whitney U test was performed for continuous variables and a Chi-squared test was performed for categorical variables.

**Table 2:** Laboratory results of study population.

Risk Factors	Patients Alive (n = 53)	Death Outcome (n = 13)	*p* Value
WBC before EE (Mean ± SD, Median [IQR])	12.041 ± 3.902, 11 [5.36]	11.438 ± 3.192, 10 [5.85]	T = 0.994
WBC on day of EE (Mean ± SD, Median [IQR])	12.466 ± 4.048, 10.9 [5.71]	12.301 ± 2.879, 10 [4.86]	0.929
WBC after EE (Mean ± SD, Median [IQR])	12.773 ± 3.902, 11.8 [6.35]	12.409 ± 2.532, 11.8 [3.65]	0.929
PLT before EE (Mean ± SD, Median [IQR])	255.49 ± 101.461, 200.1 [142]	209 ± 52.075, 195 [64]	0.124
PLT on day of EE (Mean ± SD, Median [IQR])	247.11 ± 95.082, 220.2 [116]	197.15 ± 46.737, 180 [44]	0.082
PLT after EE (Mean ± SD, Median [IQR])	245.62 ± 104.585, 210 [104]	185.62 ± 41.422, 160 [58]	0.044 *
INR before EE (Mean ± SD, Median [IQR])	1.084 ± 0.084, 1.07 [0.132]	1.186 ± 0.219, 1.10 [0.232]	0.153
INR on day of EE (Mean ± SD, Median [IQR])	1.095 ± 0.078, 1.00 [0.115]	1.158 ± 0.163, 1.10 [0.212]	0.313
INR after EE (Mean ± SD, Median [IQR])	1.087 ± 0.173, 0.90 [0.093]	1.138 ± 0.102, 1.05 [0.123]	0.116
Urea before EE (Mean± SD, Median [IQR])	5.632 ± 1.994, 5.5 [2.9]	6.554 ± 3.728, 5.8 [5]	0.741
Urea on day of EE (Mean ± SD, Median [IQR])	5.523 ± 2.131, 5.5 [2.8]	6.585 ± 3.778, 5.8 [5.5]	0.583
Urea after EE (Mean ± SD, Median [IQR])	5.370 ± 2.374, 5.0 [2.9]	6.869 ± 3.892, 6.2 [5.7]	0.269
CRP before EE (꞊or↓/↑)	13/40 (24.5%/75.5%)	4/9 (30.8%/69.2%)	0.645
WBC before EE (꞊or↓/↑)	13/40 (24.5%/75.5%)	4/9 (30.8%/69.2%)	0.645
Ly before EE (꞊or↓/↑)	47/6 (88.7%/11.3%)	13/0 (100%/0%)	0.203
PMF before EE (꞊or↓/↑)	47/6 (88.7%/11.3%)	9/4 (69.2%/30.8%)	0.080
RBC before EE (꞊or↓/↑)	51/2 (96.2%/3.8%)	13/0 (100%/0%)	0.477
HGB before EE (꞊or↓/↑)	33/0 (100%/0%)	33/0 (100%/0%)	N/A
HCT before EE (꞊or↓/↑)	52/1 (98.1%/1.9%)	13/0 (100%/0%)	0.618
PLT before EE (꞊or↓/↑)	48/5 (90.6%/9.4%)	13/0 (100%/0%)	0.249
Glucose before EE (꞊or↓/↑)	24/29 (45.3%/54.7%)	4/9 (30.8%/69.2%)	0.343
Urea before EE (꞊or↓/↑)	45/8 (84.9%/15.1%)	9/4 (69.2%/30.8%)	0.189
Potassium before EE (꞊or↓/↑)	53/0 (100%/0%)	13/0 (100%/0%)	N/A
Sodium before EE (꞊or↓/↑)	53/0 (100%/0%)	11/2 (84.6%/15.4%)	0.004
Proteins before EE (꞊or↓/↑)	47/6 (88.7%/11.3%)	13/0 (100%/0%)	0.203
PT before EE (꞊or↓/↑)	26/27 (49.1%/50.9%)	4/9 (30.8%/69.2%)	0.235
INR before EE (꞊or↓/↑)	35/18 (66%/34%)	8/5 (61.5%/38.5%)	0.760
PT% before EE (꞊or↓/↑)	48/5 (90.6%/9.4%)	13/0 (100%/0%)	0.249
APTT before EE (꞊or↓/↑)	50/3 (94.3%/5.7%)	12/1 (92.3%/7.7%)	0.783
CRP on day of EE (꞊or↓/↑)	22/31 (41.5%/58.5%)	3/10 (23.1%/76.9%)	0.220
WBC on day of EE (꞊or↓/↑)	17/36 (32.1%/67.9%)	3/10 (23.1%/76.9%)	T = 0.527
Ly on day of EE (꞊or↓/↑)	47/6 (88.7%/11.3%)	13/0 (100%/0%)	0.203
PMF on day of EE (꞊or↓/↑)	49/4 (92.5%/7.5%)	11/2 (84.6%/15.4%)	0.378
RBC on day of EE (꞊or↓/↑)	51/2 (96.2%/3.8%)	13/0 (100%/0%)	0.477
HGB on day of EE (꞊or↓/↑)	52/1 (98.1%/1.9%)	13/0 (100%/0%)	0.618
HCT on day of EE (꞊or↓/↑)	53/0 (100%/0%)	12/1 (92.3%/7.7%)	0.042
PLT on day of EE (꞊or↓/↑)	52/1 (98.1%/1.9%)	13/0 (100%/0%)	0.618
Glucose on day of EE (꞊or↓/↑)	22/31 (41.5%/58.5%)	3/10 (23.1%/76.9%)	0.220
Urea on day of EE (꞊or↓/↑)	47/6 (88.7%/11.3%)	9/4 (69.2%/30.8%)	0.080
Potassium on day of EE (꞊or↓/↑)	53/0 (100%/0%)	13/0 (100%/0%)	N/A
Sodium on day of EE (꞊or↓/↑)	53/0 (100%/0%)	11/2 (84.6%/15.4%)	0.004
Proteins on day of EE (꞊or↓/↑)	52/1 (98.1%/1.9%)	13/0 (100%/0%)	0.618
PT on day of EE (꞊or↓/↑)	30/23 (56.6%/43.4%)	6/7 (46.2%/53.8%)	0.498
INR on day of EE (꞊or↓/↑)	31/22 (58.5%/41.5%)	6/7 (46.2%/53.8%)	0.422
PT% on day of EE (꞊or↓/↑)	53/0 (100%/0%)	12/1 (92.3%/7.7%)	0.042
APTT on day of EE (꞊or↓/↑)	51/2 (96.2%/3.8%)	12/1 (92.3%/7.7%)	0.543
CRP after EE (꞊or↓/↑)	26/27 (49.1%/50.9%)	2/11 (15.4%/84.6%)	0.028
WBC after EE (꞊or↓/↑)	21/32 (39.6%/60.4%)	2/11 (15.4%/84.6%)	T = 0.100
Ly after EE (꞊or↓/↑)	48/5 (90.6%/9.4%)	13/0 (100%/0%)	0.249
PMF after EE (꞊or↓/↑)	50/3 (94.3%/5.7%)	10/3 (76.9%/23.1%)	0.050
RBC after EE (꞊or↓/↑)	52/1 (98.1%/1.9%)	13/0 (100%/0%)	0.618
HGB after EE (꞊or↓/↑)	52/1 (98.1%/1.9%)	13/0 (100%/0%)	0.618
HCT after EE (꞊or↓/↑)	53/0 (100%/0%)	13/0 (100%/0%)	N/A
PLT after EE (꞊or↓/↑)	50/3 (94.3%/5.7%)	13/0 (100%/0%)	0.380
Glucose after EE (꞊or↓/↑)	36/17 (67.9%/32.1%)	0/13 (0%/100%)	0.000
Urea after EE (꞊or↓/↑)	48/5 (90.6%/9.4%)	7/6 (53.8%/46.2%)	0.001
Potassium after EE (꞊or↓/↑)	53/0 (100%/0%)	12/1 (92.3%/7.7%)	0.042
Sodium after EE (꞊or↓/↑)	53/0 (100%/0%)	8/5 (61.5%/38.5%)	0.000
Proteins after EE (꞊or↓/↑)	47/6 (88.7%/11.3%)	13/0 (100%/0%)	0.203
PT after EE (꞊or↓/↑)	38/15 (71.7%/28.3%)	5/8 (38.5%/61.5%)	0.024
INR after EE (꞊or↓/↑)	38/15 (71.7%/28.3%)	6/7 (46.2%/53.8%)	0.080
PT% after EE (꞊or↓/↑)	53/0 (100%/0%)	13/0 (100%/0%)	N/A
APTT after EE (꞊or↓/↑)	52/1 (98.1%/1.9%)	8/5 (61.5%/38.5%)	0.000

*—statistically significant; N/A—non-applicable, Abbreviations: EE—endovascular embolization, WBC—white blood cell count, PLT—platelet count, INR—international normalized ratio, CRP—C-reactive protein; (꞊or↓/↑)—normal or not elevated/elevated, Ly—lymphocyte count, PMF—polymorphonuclear cell count, RBC—red blood cell count, HGB—hemoglobin level, HCT—hematocrit, MCV—mean corpuscular volume, MCH—mean corpuscular hemoglobin, PCT—plateletcrit, PT—prothrombin time, PT%—prothrombin activity, APTT—activated partial thromboplastin time. For the sake of clarity, the Mann–Whitney U test was performed for continuous variables and a Chi-squared test was performed for categorical variables.

**Table 3:** Univariate analysis of factors associated with intrahospital mortality.

Risk Factors	*p* Value	Crude Odds Ratio	Confidence Interval (95%)
Age	0.025	1.077 *	1.009–1.149
Gender	0.317	1.923	0.534–6.921
FS score	0.016	5.450 *	1.364–21.769
Hemorrhage in IV ventricle (yes/no)	0.046	3.590 *	1.021–12.620
Cerebral vasospasm (yes/no)	0.130	2.719	0.744–9.936
Mechanical ventilation (yes/no)	0.001	15.321 *	3.015–77.862
Hospitalization length	0.035	0.940 *	0.888–0.996
INR before EE (cont.)	0.039	468.871 *	1.367–160,817.788
CRP after EE (꞊or↓/↑)	0.041	5.296 *	1.069–26.233
Urea after EE (꞊or↓/↑)	0.004	8.229 *	1.974–34.294
Urea on day of EE (꞊or↓/↑)	0.092	3.481	0.815–14.876
PT after EE (꞊or↓/↑)	0.030	4.053 *	1.142–14.392
APTT after EE (꞊or↓/↑)	0.003	32.500 *	3.350–315.337
Urea before EE (mmol/L)	0.227	1.158	0.913–1.468
Urea on day of EE (mmol/L)	0.186	1.164	0.930–1.456
Urea after EE (mmol/L)	0.091	1.197	0.972–1.475

CRP—C-reactive protein; EE—endovascular embolization; (꞊or↓/↑)—normal or not elevated/elevated; PT—prothrombin time; APTT—activated partial thromboplastin time; INR—international normalized ratio; *—statistically significant.

**Table 4:** Multivariate analysis of factors associated with intrahospital mortality.

Risk Factors	*p* Value	Adjusted Odds Ratio	Confidence Interval (95%)
Age	0.152	1.076	0.973–1.189
Urea after EE (mmol/L)	0.036 *	12.657	1.187–134.913
Delayed ischemic neurological deficit	0.045 *	16.278	1.065–248.748
Hemorrhage in IV ventricle	0.028 *	12.128	1.307–112.575
Aneurysm size	0.476	1.709	0.392–7.460
Duration of hospitalization	0.036 *	0.916	0.844–0.994

EE—endovascular embolization; *—statistically significant.

## Data Availability

The datasets used and analyzed during the current study are made available from the corresponding author on reasonable request.
